# Immunobiologicals in dermatology^☆^

**DOI:** 10.1016/j.abd.2021.05.016

**Published:** 2022-03-18

**Authors:** Dimitri Luz Felipe da Silva, Elisa Nunes Secamilli, Mariana Valbon Beleli, Juliana Yumi Massuda, Andrea F.E.C. Franca, Renata F. Magalhães

**Affiliations:** Discipline of Dermatology, Hospital de Clínicas, Universidade Estadual de Campinas, Campinas, SP, Brazil

**Keywords:** Arthritis, psoriatic, Dermatitis, atopic, Hidradenitis suppurativa, Psoriasis

## Abstract

Immunobiologicals are a reality in current clinical practice and have increasingly gained space in the inflammatory disease scenario, especially in dermatology, with approved drugs for psoriasis, atopic dermatitis, and hidradenitis suppurativa, in addition to many others undergoing study. It is important for dermatologists to have knowledge of the medications approved in Brazil, for the best management of dermatoses, in addition to the fact that they represent hope for improvement in patients with chronic diseases.

## Introduction

Immunobiologicals are drugs produced through the biosynthesis of living cells. They are large unstable molecules with thousands of atoms and undergo changes with minimal variations in storage and conservation. They are preferably administered by injection or inhalation due to the risk of inactivation by digestive enzymes. They comprise a heterogeneous class of products developed by genetic engineering, which is associated with a high cost for the development of the technology, production, distribution, and storage.^1^

With the advancement of the knowledge of the physiopathogenesis of inflammatory diseases, the advent of target-specific therapies has produced a revolution in the treatment of immune-mediated diseases and cancer.^1^

The concept of biological medications is an old one, for instance, with the development of the serum against rabies in 1885 by Pasteur; of insulin from animal pancreas for diabetes in 1922 by Banting and Best; the extraction of growth hormone from cadavers in 1958 by Raben. With the advent of knowledge about the genome in 2000, the possibility of manipulating genes and producing specific proteins became a reality. The production of modified antibodies aimed at specific elements from the fusion of two cells (immunized mouse lymphocytes and tumor cells) in 1975 by Kohler and Milstein was the milestone in the development of monoclonal antibodies. It allowed the development of the immortalized hybridoma, a “mother cell” of a lineage that can be maintained and expanded.^1^

Groups of known protein synthesis genes can be isolated, recombined as a new gene, and transferred to a host cell that starts producing a new protein. These host cells are called an expression system, and one example is the most simple *E. coli*, or CHO (Chinese hamster ovary) cells of genetically modified mammals which are more complex. These cells are stored at low temperatures in an immortalized gene bank, and aliquots are removed for cultivation in industrial reactors with nutrients for cell growth and protein production. Subsequently, the product is recovered, separated, and purified.

They are macromolecules and can be immunogenic, with variable or unpredictable consequences, such as hypersensitivity reactions to the product, decreased efficacy due to anti-drug antibody formation, loss of immune tolerance in lupus-like syndromes.

In Brazil, several immunobiologicals are registered and recognized by the Brazilian Health Regulatory Agency (ANVISA/MoH): vaccines, hyperimmune serums, blood products, biomedicines, monoclonal antibodies, drugs containing live, attenuated, or dead microorganisms, probiotics, and allergens.

Antibodies are large heterodimeric protein molecules (molecular weight ∼150 kDa) consisting of two identical light chains and two identical heavy ones, each comprising different domains. They are joined by disulfide bonds, forming a Y-shaped structure. There are five classes of antibodies based on their heavy chain sequences: IgM, IgD, IgG, IgE, and IgA, and each class has different subtypes (IgG1, IgG2, IgG3, and IgG4). They are naturally polyclonal, i.e., produced by several clones of B lymphocytes. They specifically bind to foreign proteins, against which they were produced after sensitization. Monoclonal antibodies, in turn, are produced by a single clone of cells, in the case of hematological or autoimmune diseases, for instance, or in the case of genetically manipulated hybridomas to produce biological drugs with specific therapeutic targets. Monoclonal antibodies produced for clinical purposes are IgG because of their prolonged circulation, longer half-life, and relatively easy production.^1^

The first generation of monoclonal antibodies was mouse-derived, had lower receptor affinity, short half-lives, and was unable to deactivate the human complement system, causing intense hypersensitivity reactions.^2^

The second generation antibodies can be chimeric (contain cDNA from the murine *Fab* domain coupled to human Fc, which increases plasma half-life fivefold), humanized (replaces *Fab* and *Fc* by human proteins, with mouse antigen-binding sites), or human (consisting of recombinant elements of human antibodies).^2^

Monoclonal antibodies can have several functions, such as labeling or destroying tumor cells, inactivating enzymes, stimulating or inhibiting receptors, turning physiological functions on or off. They are useful in oncological and autoimmune treatments, transplant rejection, as diagnostic markers, and for scientific investigation.^2^

As for the nomenclature, the suffix generally expresses the nature of the product (*mab: monoclonal antibody*; *ept: fusion protein*). The middle syllable denotes the antibody source, one or two letters before the suffix (U: human, O: mouse, E: hamster; I: primate; A: rat). For example, ZU: humanized; XI: chimeric. The prefix initially denoted the disease target to be treated, but with the increasing number of new biologicals, this rule is no longer used. For example: bac (bacterial), lim (immunomodulator), vir (viral), ci (cardiovascular), mel (melanoma), pr(o) (prostate), gov (ovary), col (colon), mar (breast), got (testicles), tum (other tumors).^2^

## Tumor necrosis factor alpha (TNF-α) inhibitors

Tumor necrosis factor is a cytokine that acts on the activation of macrophages, phagosomes, differentiation of monocytes into macrophages, recruitment of polymorphonuclear cells, and granuloma formation/maintenance. It is secreted by keratinocytes and activates dendritic cells, triggering a Th1 response, which results in the production of other pro-inflammatory cytokines. It also stimulates the production of Th17 cytokines, such as IL-17 and IL-23. Thus, the inhibition of this pathway through medications has shown good responses in dermatological diseases such as plaque psoriasis (PSO) and hidradenitis suppurativa (HS), in addition to several other diseases. They modified the history of psoriasis treatment with a 75% improvement in the Psoriasis Area Severity Index (PASI) by week 16 of treatment, whereas in the prebiological era, the mean PASI was 50.^3^

Historically, it was conceived as a cachexin-inhibiting molecule for the treatment of shock and sepsis, situations in which this cytokine was high, but the studies had to be interrupted due to the increase in mortality. Some contraindications are common to TNF-α inhibitors, such as multiple sclerosis and other demyelinating diseases, optic neuritis, heart failure functional class 3 or 4, active infections, untreated latent tuberculosis, previous lymphoma or current neoplasia, congenital or acquired immunodeficiency, and hypersensitivity to the formula components.^3^

Therefore, an extensive anamnesis covering such situations is recommended, in addition to an overall laboratory screening, PPD test (or specific test, if possible), serology for hepatitis B and C, syphilis and HIV, chest X-ray, and targeted tests, if there is a suspicion in the anamnesis. It is also important to pay attention to the vaccination record updating since vaccines with live agents cannot be used during biological therapy.^3^

TNF plays an important role in fighting infections, whether viral, fungal, or bacterial, being responsible for the stability of tuberculosis caseous granuloma, as well as for the immune response restraint, which is why it is so necessary to rule out the possibility of active disease or latent infection. The same is true for patients with active surface antigen for virus B (HbsAg) when the possibility of reactivation also needs to be discussed.^4^

The risk of demyelination associated with the use of anti-TNF was raised by a drug, no longer available, called Lenercept, which would have been indicated for multiple sclerosis (MS), but led to its exacerbation, and Efalizumab, a medication used for psoriasis that was withdrawn from the market later due to cases of progressive leukoencephalopathy. Seventeen cases of etanercept-induced MS and two cases induced by infliximab were also reported. In the case of demyelinating diseases, it is postulated that there is an activation of the polyomavirus by the TNF inhibitor, triggering the disease.^4^

Neoplasm cases should be discussed directly with the assistant team, aiming to make the best decision on when to introduce the immunobiological; recent studies have shown this therapy can be considered in neoplasia cases under remission for at least five years.

TNF-alpha inhibition may be associated with exacerbation of heart failure (HF), according to randomized clinical trials of TNF-alpha inhibitors as a potential therapy for improving cardiac function in HF, whose post-marketing surveillance data collected by the FDA showed exactly the opposite: increased mortality with deterioration of cardiac function in 2001, worsening CHF, with 38 patients developing HF and nine with worsening of previous HF.^5^

The main adverse effects of TNF-alpha inhibitor drugs are cutaneous infusion reactions (local pruritus, pain, erythema and edema) with the subcutaneous administration of the drug and systemic reactions when the route is intravenous, with acute allergic reactions, anaphylaxis, fever or chronic reactions; cytopenias; infections, most commonly of the upper airways; autoimmunity induction; paradoxical psoriasis, among others.^5^

Listed below are the drugs approved in Brazil, with proven action in dermatoses, which will be discussed.

### Etanercept (Enbrel® and Brenzys™)

This drug was initially approved by the FDA for rheumatoid arthritis (RA) and psoriatic arthritis (PA), when improvement in skin lesions was observed, allowing studies with a focus on the indication for plaque psoriasis to be carried out. The drug arrived in Brazil in 2005 and had its biosimilar regulated by ANVISA in 2017.^6^

It consists of a recombinant fusion protein that binds to the p75 *Fc* fraction of human immunoglobulin G1 (IgG1), blocking the TNF-α receptor. Due to its short half-life (2–5 days) and the binding to the receptor, it is a low-antigenic and fast-acting drug, demonstrating a good safety profile. The response at week 24 was PASI 75 in 70% of patients treated with the 50 mg weekly dose and maintained a sustained response in 38% after two years, according to the pivotal studies. The pivotal studies do not consider the metric of PASI 100.^7^

Meta-analyses have shown that the drug does not decrease the risk of MACE (Major adverse cardiovascular events); however, it leads to improvement in the metabolic syndrome elements, with a decrease in abdominal circumference, triglycerides, and blood glucose levels, in addition to not affecting the glomerular filtration rate (COSTA 2014). There is no need for dose adjustment in patients with kidney and/or liver failure and the elderly. There is also the presentation in a 25 mg syringe, which can be used both for the pediatric population to facilitate the administration of the calculated dose and for the adult population if there is an indication for a decrease in the weekly dose. Studies show that the chance of changing this medication is greater due to lack of response than to its side effects.^6^

### Infliximab (Remicade® and Remsima™)

Approved for use in Crohn's disease (CD) by the FDA in 1998, this drug was approved in Brazil in 2005 and has formal indications in its package insert (Table 1). The breach of its patent in 2015 allowed the manufacture of the first Brazilian biosimilar produced at Bio-Manguinhos/Fiocruz. This drug blocks the action of circulating TNF-α, making the antibody bound to the cytokine unable to bind to its receptor or to activate it, in addition to participating in the lysis of cells that express it on the surface activating the complement system.^5^

**Table 1:** Medications for psoriasis.

Medication	Action mechanism	Half-life	Indication (package insert)	Presentation	Dosage
Etanercept	Anti-TNF-α	3 days	PSO (adults and children ≥8 years), RA, PA, AS and axial AS	Pre-filled syringe, 25 and 50 mg	I: 50 mg SC, 2× week for 12 weeks;
					M: 50 mg SC, 1× week a
Infliximab	Anti-TNF-α	25 days	PSO, PA, RA, AS (adults); CD and RU (adults and ≥ 6 years)	Ampoule vial with lyophilized powder 100 mg	I: 5 mg/kg IV, weeks 0-2-6
					M: 5 mg/kg IV 8/8 weeks
Adalimumab	Anti-TNF-α	14 days	PSO, PA, HS, RU, RA, AS, axial AS (adults); CD, Uveitis, JIA and ERA (adults and children ≥ 2 years)	Syringe, ampoule vial and pen, 20, 40 and 80 mg	I: 80 mg D0, 40 mg D7,
					M: 40 mg every 14 days b
Certolizumab-pegol	Anti-TNF-α	14 days	PA, CD, RA and AS	Pre-filled syringe, 200 mg	I: 400 mg SC, weeks 0-2-4;
					M: 200 mg SC, every 2 weeks or 400 mg SC, every 4 weeks c
Ustekinumab	Anti-IL-12,23	10 weeks	PSO, PA, CD	Syringe and ampoule vial, 45 and 90 mg	I: 45 mg SC weeks 0-4;
					M: 45 mg SC every 12 weeks d
Secukinumab	Anti-IL-17	27 days	PSO, PA and AS	Pre-filled pen, 150 mg	I: 300 mg SC weeks 0–1–2–3–4;
					M: 300 mg SC every 4 weeks
Ixekizumab	Anti-IL-17	13 days	PSO	Pre-filled pen, 80 mg	I: 160 mg SC weeks 0, 80 mg weeks 2–4–6–8–10–12;
					M: 80 mg SC every 4 weeks
Guselkumab	Anti-IL-23	17 days	PSO; PA	Pre-filled syringe, 100 mg	I: 100 mg SC weeks 0–4;
					M: 100 mg SC every 8 weeks
Risankizumab	Anti-IL-23	4 weeks	PSO	Pre-filled syringe, 75 mg	I: 150 mg SC weeks 0–4;
					M: 150 mg SC every 12 weeks

a Etanercept pediatric dose– 0,8 mg/kg (max. 50 mg) SC, 1× week.

b Adalimumab par HS – I: 160 mg D0, 80 mg D14, 40 mg D28; M: 40 mg every 7 days.

c Dose corresponding to psoriatic arthritis.

d In patients weighing more than 100 kg, the dose should be 90 mg; Pediatric used from 9 years of age on.

As this is an intravenous administration drug, results can be achieved more quickly, but the incidence of side effects is also higher. The most common side effects are related and can occur up to two hours after the infusion and should be monitored in a hospital environment. Regarding efficacy, studies show a PASI 75 at week 10 for 72% of the patients using 3 mg/kg, and 88% for those using the 5 mg/kg dose, with PASI 90 being observed in 58% of subjects using this dose; in comparison, only 42% reach PASI 75 when using MTX.^5^, ^6^

On the other hand, safety studies after one year of drug use show that the administration should be constant and within the recommended interval since intermittent use increases the risk of production of anti-drug antibodies and, consequently, loss of secondary response.^5^

In safety studies, 64% had at least one mild adverse effect (as previously highlighted), with infusion reactions, nasopharyngitis, fatigue, and nausea being the most common ones, with only 5% of patients showing severe infections or cardiovascular manifestations. Studies of 20 years of drug use show that this profile has remained stable, but there is a greater induction of ANA positivity and drug-related lupus (9% cases), therefore hindering its use in chronic infections, even when controlled.^5^

### Adalimumab (Humira® and Amjevita™)

Of the drugs presented in this article, this is the one with the widest range of indications, as well as the greatest use in the world population. It was approved by ANVISA in 2008, and the manufacturing of its biosimilar was approved in 2019. It is also the first fully human monoclonal antibody on the list, which acts by blocking circulating TNF-a by binding to the IgG1 region. With subcutaneous administration, it is, to date, the only drug with formal indication, also found in the package insert (Table 1) for two dermatoses: psoriasis and hidradenitis suppurativa, with the latter being regulated in 2017.^6^

The efficacy is demonstrated with 78.3% of patients treated for plaque psoriasis reaching PASI 75 at week 16, with first responses appearing as early as at week four and demonstration of a sustained response with PASI 75 in 50.3% of patients at week 52. Pivotal studies do not consider the metric of PASI 100.^7^

Regarding hidradenitis, studies show a 50% improvement in HiSCR (Hidradenitis Suppurativa Clinical Response score) after 12 weeks of treatment; however, data on a sustained response are lacking.^8^

The pivotal studies show a good safety profile, with an incidence of side effects similar to the use of placebo, with the majority being mild or moderate, such as pain or erythema at the injection site and upper airway infections. One case of TB was reported in a patient with previous negative PPD, but after being retested, there was a “change” and the medication was discontinued. Congestive heart failure was demonstrated in only one patient in the REVEAL study. Despite using higher doses and shorter intervals, studies have not shown a higher incidence of side effects, both mild and severe, in patients treated for HS.^6^, ^8^

### Certolizumab pegol (Cimzia®)

Regulated by ANVISA in 2019, initially with the indication for RA and PA, this drug also has an effect on plaque psoriasis and acts by inhibiting circulating TNF-a levels. It is a molecule that shows a covalent binding of the antigen to the monoclonal antibody (Fab' fragment) to a polyethylene glycol molecule; this PEGylation process reduces the antigenicity and prolongs the half-life of the agent. The molecule does not include an *Fc* portion and does not induce complement activation, in addition to not binding to IgG, the only cytokine that crosses the placental barrier, which is why it is the only safe biological drug for administration during pregnancy.^9^

Its efficacy was demonstrated with PASI 75 at week 12, being 75% for those using 200 mg and 83% for 400 mg. In retreatment studies, the results at week 12 were similar in both groups. The drug has a good safety profile, with gastrointestinal and upper airway infections being the most common adverse effects. The pivotal studies do not include the metric of PASI 100. The incidence of MACE, malignancies, opportunistic infections or death was less than 1%, consistent with the general population. The difference in dosage also did not seem to influence a greater expression of side effects.^10^

## Interleukin 12,23 (IL-12,23) inhibitor

IL-12 and IL-23 are heterodimeric cytokines secreted by activated antigen-presenting cells, such as macrophages and dendritic cells. IL-12 stimulates natural killer (NK) cells and conducts the differentiation of CD4+ T-cells into the T Helper 1 (Th1) cell phenotype, and stimulates the production of interferon-gamma (IFNγ). IL-23 induces the T17 helper cell (Th17) pathway and promotes the secretion of IL-17A, IL-21, and IL-22. The levels of IL-12 and IL-23 are elevated in the skin and blood of patients with psoriasis, and serum IL12/23p40 differentiates between patients with psoriatic arthritis and healthy individuals, implicating IL-12 and IL-23 in the pathophysiology of psoriatic inflammatory diseases.^11^

IL-23 responsive T cells were found in enthesis in a mouse model of inflammatory arthritis, where IL-23 conducts the inflammation of the enthesis. Additionally, there is pre-clinical evidence implicating IL-23 and descending pathways in bone erosion and bone destruction through the increase in the receptor activator of nuclear factor-kappa B ligand (RANKL), which activates osteoclasts. ^12^

Ustekinumab is a fully human IgG1kappa monoclonal antibody that specifically binds to the p40 shared protein subunit of the human cytokines interleukin 12 (IL-12) and IL-23. Ustekinumab inhibits human IL-12 and IL-23 bioactivity by preventing p40 from binding to the IL-12Rbeta1 protein receptor expressed on the surface of cells from the immunological system.^11^, ^12^

Approved in Brazil in 2012, the drug has a good safety profile, whose main adverse effects are upper airway infections, local reactions, diarrhea, nausea, and pruritus. Its contraindication is hypersensitivity to the drug components, and it seems to have no action on axial psoriatic arthritis; however, it has a good response in dactylitis and peripheral arthritis. The dose should be adjusted to 90 mg in individuals weighing more than 100 kg.^13^

The drug showed a sustained response in a 5-year follow-up study since the first pivotal study (PHOENIX 1 and 2) and resulted in PASI 75 in approximately 77% of patients, and PASI 90 in 50%, at week 12. The pivotal studies do not contemplate the metric of PASI 100.

Nevertheless, real-life studies show that the criteria used in the pivotal studies are far from the reality of patients who actually use the drug and that the rate of side effects is slightly higher in the real-life population.^14^

The drug has shown a good safety profile when compared to methotrexate and other biological drugs, not increasing the risk of MACE, malignancies, or sudden death. There are some case reports in the literature demonstrating the drug is safe to be used in concomitant chronic infections, such as HIV and hepatitis, without reactivation of viral repplication.^11^, ^12^, ^13^

## IL-17 inhibitors

As a product of the stimulation of dendritic and antigen-presenting cells by pro-inflammatory cytokines, what is currently generically known as the “type 3 response” is triggered, the Th17 response, with the production of IL-23, which is later transformed into IL-17 and its subunits, with A and F subunits being the most frequently involved in the physiopathogenesis of psoriasis.

This cytokine plays a role in the production of other microbial cytokines and peptides, with the recruitment of neutrophils and polymorphonuclear cells, stimulating the production of IL-1-β and TNF-α through the recruitment of these cells, in addition to contributing to the Th2 response against extracellular organisms. The IL-17 family includes cytokines IL-17A, IL-17B, IL-17C, IL-17D, IL-17E (also referred to as IL-25), and IL-17F.^15^

In addition to the recruitment of neutrophils, it has an important mucocutaneous presence, which may be related to an increase in oral and genital candidiasis, mostly in mild or moderate cases. It is important to pay attention to this in patients with inflammatory bowel disease (IBD – Crohn's disease and ulcerative colitis) since there are reports of worsening of these diseases during the treatment. However, there are no data to support the conclusion that these drugs induce IBD.^16^, ^17^

### Secukinumab (Cosentyx®)

It is a fully human IgG1k monoclonal antibody, which selectively inhibits the binding of IL-17A and F with their receptors. It arrived in Brazil in December 2015, initially with approval for use in plaque psoriasis, later gaining the indication for use in PA and AS, as well.^15^

Among the main side effects, approximately 1% of patients in pivotal studies had oral herpes, nasopharyngitis, URIs, diarrhea, and urticaria, being a drug with a good safety profile, except for the aforementioned situations.^18^

The randomized placebo trials ERASURE and FIXTURE demonstrated the effectiveness of secukinumab at a monthly dose of 300 mg for moderate to severe plaque psoriasis, with PASI 75 in 82% of patients at week 12, when compared to placebo or the lower monthly dose of 150 mg, and PASI 100 in 24% of patients at week 12.^19^

The CLEAR study, with bolder endpoints, started from the comparison with ustekinumab and demonstrated a PASI of 90 in 79% of patients at week 16 and 76% at week 52.^20^

Regarding special areas, there are real-life studies showing clearance of the ppPGA index (for palmoplantar psoriasis) and a reduction of more than 90% of the scalp PGA (affected scalp area). The TRANSFIGURE study showed a mean reduction in the Nail Psoriasis Severity Index (NAPSI) of 73.3% at week 16, with studies showing a sustained response for 2½ years for ungual psoriasis.^20^

### Ixekizumab (Taltz®)

Approved by ANVISA in 2017, it is a humanized IgG4 antibody, which decreases the levels of IL-17A and F, IL-22 and IL-23 through its high affinity for IL-17A, binding to the circulating portion.^21^

Its main reported side effects are injection site reactions, URIs, neutropenia, candidiasis, and IBD, showing a good safety profile for more severe reactions, most of which are transient and do not require drug discontinuation (DYRING 2015).^22^

The UNCOVER studies 1, 2, and 3 are the pivotal studies that compared ixekizumab at two different doses with placebo and etanercept, leading to the conclusion that the safe and effective dose for the treatment of moderate to severe plaque psoriasis is 80 mg 1x month, after induction with 160 mg. A PASI 75 was demonstrated in 90% of those treated at week 12, maintaining a sustained response in up to 74% of those treated at week 60. Other studies have shown that patients reached, at the end of week 12, PASI 75 in 98.7%, PASI 90 in 83.3% of cases, and PASI 100 in 41%.^23^, ^24^

## IL-23 inhibitors

IL-23 is responsible for inflammation, immune activation, and hyperproliferation of the stratum corneum. It is a heterodimer consisting of two subunits: p40, which is common to IL-12 in the Th1 pathway, and p19, which is specific only for IL-23. Messenger RNA levels of both the p19 and p40 subunits for IL-12/IL-23 are increased in psoriasis plaques and decrease during the treatment.

The expression of retinoic acid-related orphan receptors (RORs) activates the production of IL-23 by APCs (antigen-presenting cells) and myeloid dendritic cells, which will act on the entire Th17 and Th22 axis and can transform into effector response cells resident in the affected plaque, even when there is clinical resolution.^25^

### Guselkumab (Tremfya®)

It is a human IgG1λ monoclonal antibody, which acts by inhibiting the secreted IL-23 and preventing the binding of circulating IL-23 to its receptors, present on the cell surface, through the specific blocking of the p19 subunit, leading to a decrease in serum, skin, and joint levels of IL-17-A.^26^

This molecule arrived on the Brazilian market in 2018 with the indication for use in plaque psoriasis, gaining, in 2019, the indication for use in psoriatic arthritis as well. With a good safety profile, the most common side effects are those already known for other biologicals, with no reported cases of severe cardiovascular events, malignancies, or generalized infections.^27^

There are several pivotal and head-to-head studies on this drug, particularly the VOYAGE 1 and 2, which show the comparison with adalimumab and placebo, respectively. A PASI 90 is seen at week 16 in 73% of patients and a PASI 100 in 37.4%, and this average increases when assessed at week 48.^28^

When compared to ustekinumab, the NAVIGATE trial showed an advantage in switching patients who did not respond to ustekinumab, ultimately responding when switched to guselkumab. In the ECLIPSE study, it was shown to be superior to secukinumab, demonstrating PASI 90 in 84.5% of the patients treated with guselkumab and PASI 100 in 58.2%, both at week 48.^26^, ^27^, ^28^, ^29^

### Risankizumab (Skyrizi®)

Approved by ANVISA in 2019. Brazil approved it simultaneously with other developed countries, such as Japan. It is a humanized monoclonal antibody that binds to the p19 subunit of IL-23.^30^

The drug has side effects such as local reactions and URIs, showing a good safety profile in the pivotal studies. These studies compared the drug with placebo and ustekinumab (UltiMMa 1 and 2), both demonstrating PASI 90 in 75% of subjects at the end of week 16, whereas 51% showed a PASI 100 response. (GORDON 2018, AL-JANABI 2019). The phase 3 IMMvent study demonstrated the superiority of risankizumab in relation to adalimumab, with 72% achieving PASI 90 versus 47% in patients in the adalimumab arm at the end of week 16.^31^

## Interleukins 4, 13 (IL-4, IL13) inhibitor

Interleukins 4 (IL-4) and 13 (IL-3) are, together with IL-5, the main effectors of the Th2 immune response. They are produced by cells of innate and adaptive immunity, have antiparasitic action, and are involved in several processes that can culminate in allergic diseases, such as a) Recruitment and activation of Th2 cells, eosinophils, and mast cells; b) B lymphocyte class switch for IgE production; c) Mechanisms involved in pruritus (such as IL-31 production and sensory neuron signaling); in addition to d) Tissue remodeling, fibrosis, and induction of defects in the cutaneous and mucosal barrier. Among the diseases triggered by activation of Th2 immunity are atopic dermatitis (AD), allergic asthma, nasal polyposis, and eosinophilic esophagitis.^32^, ^33^

A part of the common functions of IL-4 and IL-13 is due to the presence of the IL-4 receptor alpha subunit (IL4Rα), which is shared by the type 2 receptor complex (of IL-4 and IL-13), present in epithelial cells, smooth muscle cells, fibroblasts, monocytes, and activated B cells.^33^

**Dupilumab** (Dupixent®) is a fully human IgG4 monoclonal antibody that inhibits IL-4 and IL-13 signaling by binding to the IL4Rα subunit of type 1 and type 2 receptors (common to both cytokines) (Table 2).

**Table 2:** Other biological medications.

Medication	Action mechanism	Half-life	Indication (package insert)	Presentation	Dosage
Omalizumab	Anti-IgE	26 days	CSU, allergic asthma	Ampoule-vial with lyophilized powder 100 mg	300 mg SC every 4 weeks
Dupilumab	Anti-IL-4, IL-13	7 days	Moderate to severe AD	Pre-filled syringe, 300 mg	I: 600 mg SC, without 0
					M: 300 mg SC, every 2 weeks
Rituximab	Anti- B Lymphocytes	3 weeks	Moderate to severe PV, RA, Leukemia, Lymphomas, Vasculitis	Ampoule-vial with lyophilized powder 100 mg/10 mL or 500 mg/50 m L	2 doses of 1000 mg with a 14-day interval^a^

a Dose recommended for RA.

It is the first FDA-approved immunobiological for severe atopic dermatitis in adults (2017), followed by its approval for adolescents aged 12 to 17 (2019) and children aged six years and older (2020). Its approval in Brazil follows the same indications. There was considerable improvement in pruritus in all studies, comparable to placebo. At week 16, peak pruritus was significantly reduced by about 1.1–3.2 points from the initial score, also improving quality of sleep in the Dermatology Life Quality Index (DLQI), in addition to significantly reducing anxiety and depression symptoms.^33^, ^34^

The pivotal SOLO 1 and 2 studies, in patients with moderate AD without improvement with topical measures (including topical corticosteroids and calcineurin inhibitors), achieved EASI 75 (75% improvement from the initial EASI – Eczema Activity Severity Index) and 48% after 16 weeks of use. This percentage increased to 62% when the use of medium-potency topical corticosteroids was associated (CHRONOS study), an effect maintained for 52 weeks and also seen in more severe patients with cyclosporine indication (CAFÉ study). There was considerable improvement in pruritus in all studies, comparable to placebo.^34^

The common side effects in all studied populations were injection site reaction (the administration is subcutaneous), headache, and conjunctivitis. Also included in this group are allergic, viral, bacterial conjunctivitis, and atopic keratoconjunctivitis. It is important to emphasize that conjunctivitis was not a more frequently observed adverse effect when compared to placebo in dupilumab studies in other Th2-mediated diseases, such as asthma and chronic rhinosinusitis. The etiology of this adverse effect remains uncertain. There was a decrease in skin infections (with the exception of herpes simplex) and AD flare episodes in the dupilumab groups.^35^

## Immunoglobulin E (IgE) Inhibitor (Xolair®)

**Omalizumab** is a humanized anti-IgE monoclonal antibody, which binds to the C3 domain of the IgE heavy chain and prevents binding to the mast cell high-affinity IgE receptor (FcεRI). The medication binds to free IgE, promoting a decrease in the expression of IgE receptors on the cell surface, preventing mast cell degranulation. It was approved in 2014, with an indication for the treatment of chronic spontaneous urticaria for individuals aged 12 years and older.^36^

The pivotal ASTERIA I and II and GLACIAL studies showed control of urticaria (urticaria activity in 7 days equal to 0 and ≤ 6) in at least 85% of patients compared to placebo (use of a standard dose of antihistamines up to 4× the package insert dose) at week 12, an effect maintained until the end of the study (week 24). Regarding pruritus, the main symptom of urticaria, there was a 71% reduction in the evaluated scores. Real-life experiences in a Brazilian sample achieved complete response (no wheals and no pruritus) in 84% of the patients.^36^, ^37^

To assess the individual response, it is recommended to maintain the use of omalizumab for six months, as there are fast and slow responders. A progressive increase in treatment response was observed in up to 24 weeks. The treatment should be maintained until complete control of wheals and pruritus (UAS7 = 0).^38^

The most frequent adverse effects correspond to headache and upper airway infection with the medication use, and tests prior to the treatment are not necessary. There was no increase in infestations or parasitosis.

## **Anti-B Lymphocytes** (MabThera®; Riximyo®)

On 10/29/2019, ANVISA approved its indication for use in moderate to severe pemphigus vulgaris (PV). Rituximab is a humanized chimeric monoclonal antibody directed against the CD20 antigen on B lymphocytes. It works by depleting circulating B lymphocytes for six to 12 months. The indications in the package insert in Brazil include chronic lymphoid leukemia, non-Hodgkin lymphoma, rheumatoid arthritis, and granulomatosis with polyangiitis. In dermatological diseases, therefore, its use is off-label.

The therapeutic effectiveness of this drug has already been evaluated in a series of cases, randomized clinical trials, and meta-analyses. Its administration is through the intravenous route and there are two dosage possibilities: as used in rheumatoid arthritis, that is, 1000 mg in two doses (0 and 15 days), in addition to 500 mg maintenance doses (months 12 and 18) or as used in lymphoma, at a dose of 375 mg/m^2^/week for four weeks, usually associated with the use of systemic corticosteroids.^39^

In the RITUX 3 study, in which rituximab plus a short course of prednisone was compared to the use of prednisone alone as first-line treatment, it was possible to demonstrate a higher rate of complete remission off therapy at 24 months (89%×34%), in addition to a steroid-sparing effect with a favorable safety profile in the group treated with rituximab. There is no consensus on the optimal dose of rituximab; a retrospective cohort study found a greater chance of complete remission in patients who submitted to the regimen used in lymphoma cases, but more studies are necessary.^40^

Moreover, there are studies in nodal lymphomas that compare the intravenous use of rituximab to its subcutaneous use, in which the subcutaneous administration has the same efficacy as the intravenous one, with a higher rate of patient satisfaction; its use in PV is also off-label. In general, rituximab is used together with systemic corticosteroids.^41^

Other autoimmune diseases can also be treated off-label with rituximab. A meta-analysis highlights bullous pemphigoid, cryoglobulinemia, and IgG4-related diseases as autoimmune diseases showing promising results to the drug use. (Kaegi 2019) In addition to autoimmune diseases, rituximab can be used in cutaneous B-cell lymphomas. There are no randomized studies published yet, but the guidelines of the European Organization for Research and Treatment of Cancer (EORTC), the International Society for Cutaneous Lymphomas, and the National Comprehensive Cancer Network indicate rituximab alone as an option for generalized cutaneous forms of primary centrofollicular and marginal zone lymphoma of the skin.^39^

Rituximab is contraindicated in patients with hypersensitivity to the active substance or the murine proteins, patients with active, severe infections, severely immunocompromised patients, patients with severe heart failure (class IV), or severe uncontrolled heart disease. This medication should not be used in pregnant and lactating women. The adverse effects include hypersensitivity infusion reactions, cardiovascular events (angina, cardiac arrhythmia, or myocardial infarction), in addition to the potential increased risk of infections, including progressive multifocal leukoencephalopathy and hepatitis B reactivation.^42^

## Financial support

None declared.

## Authors’ contributions

Dimitri Luz Felipe da Silva: Approval of the final version of the manuscript; design and planning of the study; drafting and editing of the manuscript; collection, analysis, and interpretation of data; effective participation in research orientation; critical review of the literature; critical review of the manuscript.

Elisa Nunes Secamili: Drafting and editing of the manuscript; collection, analysis, and interpretation of data; critical review of the manuscript.

Mariana Valbon Beleli: Drafting and editing of the manuscript.

Juliana Yumi Massuda: Collection, analysis, and interpretation of data.

Andrea F. E. C. Franca: Drafting and editing of the manuscript; collection, analysis, and interpretation of data; critical review of the literature.

Renata F. Magalhães: Approval of the final version of the manuscript; design and planning of the study; drafting and editing of the manuscript; collection, analysis, and interpretation of data; effective participation in research orientation; critical review of the literature; critical review of the manuscript.

## Conflicts of interest

Dimitri Luz Felipe da Silva, Juliana Yumi Massuda Andrea F. E. C. Franca and Renata F. Magalhães declare they have conflicts of interest regarding the following companies: Lilly, Novartis, Abbvie and Janssen.
